# Chronic granulomatous disease associated with systemic lupus erythematosus and systemic onset juvenile idiopathic arthritis

**DOI:** 10.1186/1546-0096-12-S1-P169

**Published:** 2014-09-17

**Authors:** Vadood Javadi Parvaneh, Reza Shiari, Leila Mahbobi, Delara Babaei

**Affiliations:** 1 Pediatric Rheumatology, Mofid Children's Hospital, Tehran, Iran, Islamic Republic Of; 2 Pediatric Immunology, Mofid Children's Hospital, Tehran, Iran, Islamic Republic Of

## Introduction

Chronic granulomatous disease (CGD) is a primary immune deficiency that characterized with recurrent infections and granuloma formation. A variety of autoimmune disorders and immune-mediated phenomena such as inflammatory bowel disease, discoid lupus erythematous, juvenile idiopathic arthritis, Ig A nephropathy, sarcoidosis, rheumatoid arthritis, idiopathic thrombocytopenia, eosinophilic cystitis, celiac disease, autoimmune hemolytic anemia and antiphospholipid syndrome have been associated with CGD.

## Objectives

Herein we reported two cases of CGD associated with systemic lupus erythematous and systemic onset juvenile idiopathic arthritis. The first case was a 13-years-old girl with previous history of tuberculosis and recurrent fever and the second one was a 3.5- years-old boy with continuous high spiking fever and arthritis.

## Methods

A 13-years-old girl with previous history of tuberculosis and recurrent fever. Her more evaluation revealed multi focal spleen granulomatosis and the result of nitro blue tetrazolium test (NBT) was zero. Malar Rash, photosensitivity, pancytopenia, and splenomegaly observed on clinic and her para clinic evaluation showed very high level of antinuclear antibody (ANA), anti double strand DNA (ds-DNA), and low levels of complements C3 and C4.

The second case was a 3.5- years-old boy with continuous high spiking fever and arthritis. He was previously diagnosed as CGD. However his more evaluation guided us to diagnosis of systemic onset juvenile idiopathic arthritis associated with CGD.

## Results

Primary immunodeficiency diseases may present with autoimmunity as well as severe, recurrent, or unusual infections.

## Conclusion

Recognizing and appropriately managing autoimmune complications in patients with underlying immunodeficiencies require an understanding of which compartment of the immune system is most affected by a patient’s particular disorder.

## Disclosure of interest

None declared.

